# 991. Single Center, Exploratory, Open-label Prospective Study Using the Minimally Invasive LysinDAIR Procedure (Lysin Administration During an Arthroscopic DAIR Procedure) in Patients with Suspected Relapsing Chronic Coagulase-Negative Staphylococci (CNS) Prosthetic Hip Infection (PHI)

**DOI:** 10.1093/ofid/ofac492.833

**Published:** 2022-12-15

**Authors:** Tristan Ferry, Anne Conrad, Camille Kolenda, Frederic Laurent, Axel Schmidt, Cara Cassino, Sebastien Lustig, Cécile Batailler

**Affiliations:** Hospices Civils de Lyon, Lyon, Rhone-Alpes, France; Hospices Civils de Lyon, Lyon, Rhone-Alpes, France; Hospices Civils de Lyon, Lyon, Rhone-Alpes, France; Hospices Civils de Lyon, Lyon, Rhone-Alpes, France; Hospices Civils de Lyon, Lyon, Rhone-Alpes, France; Contrafect Corporation, New-York, New York; Hospices Civils de Lyon, Lyon, Rhone-Alpes, France; Hospices Civils de Lyon, Lyon, Rhone-Alpes, France

## Abstract

**Background:**

Exebacase, a recombinant staphylococcal lysin, has: (i) reported proof-of-concept data in Phase II in *S. aureus* bacteremia; (ii) demonstrated antibiofilm activity *in vitro* against *S. epidermidis;* and (iii) been used as salvage therapy in 4 patients with relapsing multidrug-resistant (MDR) *S. epidermidis* knee prosthetic joint infection (PJI) using the LysinDAIR (arthroscopic administration of exebacase [Lysin] with Debridement and Antibiotics Implant Retention) procedure. Surgical treatment of recurrent PHIs has high risk of morbidity and loss of function. We now report our experience with the LysinDAIR procedure for PHIs.

**Methods:**

We performed a single center, exploratory, open-label prospective study using LysinDAIR and suppressive antimicrobial therapy (SAT) in patients with recurrent, chronic (inoculation >3 months prior to treatment) CNS PHI. In agreement with the French Health authority, exebacase (2 to 3.5 total mg in 30-50 ml [∼0.067 – 0.075 mg/ml]) was arthroscopically administered directly into the joint.

**Results:**

Three consecutive patients were included (55 to 75 years). All had previous iterative hip prosthesis exchange with a high total number of surgeries (from 6 to 10) and recent persistent or intermittent fistula (Figure). All refused hip prosthesis explantation or surgeon considered explantation too high risk for loss of function and severe post-operative complications. Patients A and B had MDR *S. epidermidis*; Patient B had *P. aeruginosa* co-infection at the time of LysinDAIR. Patient C had a prior persistent *S. lugdunensis* PHI and relapse on arthrocentesis; *K. pneumoniae* was cultured at the time of LysinDAIR. No adverse events related to exebacase or the procedure were reported. At 2 years follow up, all patients had resolution of fistula and no clinical signs of infection on SAT (tedizolid (+/- ciprofloxacin [Patient B] or cotrimoxazole [Patient C]).

Description of the patients treated with the LysinDAIR procedure

**Figure:**
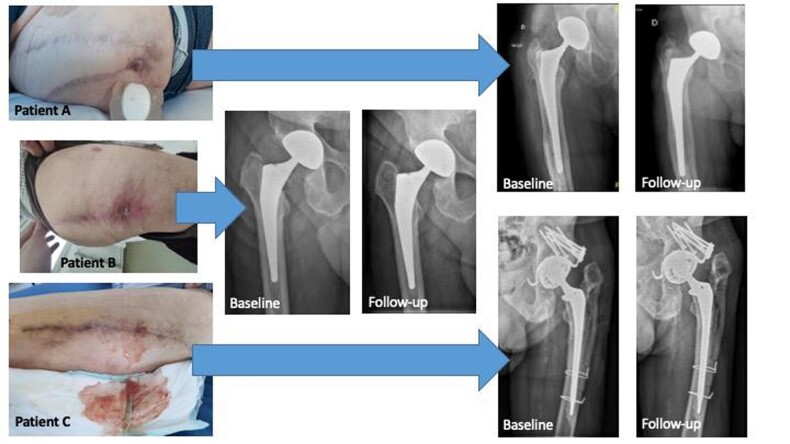

Local signs of infection with discharge of the three patients (A, B and C) with complex relapsing prosthetic hip infection with X-ray before the LysinDAIR procedure and at 2 years of follow-up

**Conclusion:**

The LysinDAIR procedure was easy-to-perform, safe and may have therapeutic potential to facilitate the success of SAT salvage therapy for chronic relapsing CNS PJIs, and potential cure of initial chronic PJI. Randomized clinical trials evaluating the LysinDAIR procedure in these clinical situations are clearly warranted.

**Disclosures:**

**Tristan Ferry, MD, PhD**, Contrafect: Advisor/Consultant **Camille Kolenda, n/a**, Contrafect: Research grant **frederic Laurent, n/a**, Contrafect: Research grant **cara cassino, n/a**, Contrafect: Employee.

